# Radionuclide uptake by colloidal and particulate humic acids obtained from 14 soils collected worldwide

**DOI:** 10.1038/s41598-018-23270-0

**Published:** 2018-03-19

**Authors:** Peng Lin, Chen Xu, Wei Xing, Luni Sun, Daniel I. Kaplan, Nobuhide Fujitake, Chris M. Yeager, Kathleen A. Schwehr, Peter H. Santschi

**Affiliations:** 1 Department of Marine Science, Texas A & M University at Galveston, Galveston, Texas 77553 United States; 2 Savannah River National Laboratory, Aiken, South Carolina, 29808 United States; 3 Division of Agroenvironmental Biology, Graduate School of Agriculture Science, Kobe University, Kyoto, 606-8501 Japan; 4 Los Alamos National Laboratory, Los Alamos, New Mexico, 87545 United States

## Abstract

Uptake of six particle-reactive and/or redox-sensitive radionuclides (^210^Pb, ^234^Th, ^7^Be, ^59^Fe, ^237^Np and ^233^Pa) by 14 humic acids (HAs) was investigated in artificial groundwater under mildly acidic conditions (pH~5.5). In HA-groundwater slurry, Pb, Be, Fe and Pa bound strongly to particulate HA (>0.45 µm), supporting their application as tracers of soil erosion. Th bound strongly to the colloidal HA (3 kDa-0.45 µm) and as such, would not be a good candidate as a tracer for monitoring soil erosion. HAs likely reduced the oxidized neptunyl form (Np(V)O_4_^+^) to Np(IV) based on its enhanced particle-reactivity and Np uptake by particulate HAs, partially retarding the movement of anthropogenic ^237^Np in field polluted environments. Particulate/colloidal carbonyl/O-aryl (likely through hydroquinone/quinone) functionalities in the HA correlated to Np and Pa uptake, but only particulate O-aryl functionalities was responsible for Fe uptake. The carboxylate- and carbonyl/O-aryl-containing organic functionalities in the HA correlated strongly with Th uptake. In contrast, no significant correlations between organic parameters and Pb or Be uptake implied their predominance of uniform surface adsorption onto particles. This study provides novel insight into the binding of six radionuclides with different organic functionalities of three size fractions, as well as its possible impact on their application in the soil-tracing research.

## Introduction

Environmental radionuclides from either natural or anthropogenic sources participate in many physical and geochemical processes in terrestrial and aquatic environments and thus can serve as good tracers of these processes. With different half lives and chemical properties, radionuclides in natural environments will have different residence times and interactions with natural particles and surrounding waters. One of the most important characteristics of a tracer for it to be used to estimate the rate of particle movement in aquatic and terrestrial systems is that it must be highly particle-reactive^1–5^. Several metals and isotopes have been used in this manner, including natural actinides (e.g., thorium, Th; protactinium, Pa) and metals (e.g., lead, Pb; beryllium, Be). Additionally, artificially labeling the soil particles with anthropogenic radionuclides, such as ^59^Fe (half-life 45 days) makes the determination of the extent and the source of soil loss possible^6^. However, strong particle-reactivity could also result in strong affinity to natural colloidal organic matter (COM). COM is usually operationally defined by the membranes used to separate it from the truly dissolved fraction. COM is composed of large organic molecules that can bind metals/tracers. The COM fraction, by virtue of size and unique chemistry of this fraction, can be released from the immobile soil phase during flooding events and has been shown to be responsible for enhanced transport of radionuclides^7–11^. As such, a potential artifact associated with using tracers to estimate soil particulate movement is that tracers associated with the mobile colloidal fraction may indicate greater mobility than in fact occurs with the soil particles. This compromises the use of tracers for their intended purpose of estimating such geochemical processes as soil/sediment erosion.

Furthermore, some radionuclides undergo redox changes upon interacting with OM that alters their particle-reactivity (e.g., plutonium, Pu(V) to Pu(IV); protactinium, Pa(V) to Pa(IV); neptunium, Np(V) to Np(IV))^12–14^. Previous studies have demonstrated that organic-rich soils strongly influence the oxidation state of some radionuclides and thus their mobility in soil environments. For example, the reduction of Pu(V) to Pu(IV) is facilitated in the presence of humic substances and by subsequent chelation of Pu(IV) to highly reactive and specific siderophore moieties in COM^15^.

Therefore, natural organic matter (NOM) in soil environments can play diverse roles related to the particle-reactivity and redox activity of radionuclides. Furthermore, NOM in the colloidal form, may promote movement of radioisotopes, while NOM in the particulate form or bound to soils, may promote radioisotope immobilization and thereby are useful for the intended purpose of soil-tracing studies.

In the present study, a laboratory experiment was conducted to examine the partitioning and uptake of six different radionuclides in the SOM-groundwater slurry, including ^210^Pb, ^234^Th, ^237^Np, ^233^Pa, ^7^Be and ^59^Fe, as representative of particle-reactive and/or redox-sensitive radionuclides. The objective of the work was to evaluate the partitioning of various radionuclides to a wide range of NOM and to evaluate the tendency of these radionuclides to partition into colloidal or truly dissolved fractions. The second objective was to correlate radionuclide uptake to various chemical characteristics of the particulate, colloidal, and truly dissolved NOM fractions. The intent of this research was to evaluate whether radionuclide partitioning to the COM fraction may compromise the use of this technique for tracking soil/sediment accumulation and erosion. The study was also designed to provide insights into the binding ability of different radionuclides to different types of humic acid substances (HAs) with widely varying composition. HAs were chosen as representative of SOM since HAs are not only the major components in the SOM pool but also have redox ability with diverse organic functional groups. To purify HAs, various types of soil, with widely varying composition, from widespread locations were selected, including Luvisols, Chernozems, Podozol, Andosols, Histosols and Cambisols, originating from different countries, including United States, Brazil, Hungary, Tanzania, Scotland and Japan (Table S1). The HAs were characterized by ^13^C nuclear magnetic resonance (NMR) spectroscopy. The wide range of organic matter sources (biota) and various weathering conditions resulted in different molecular and elemental compositions of purified HAs, with contrasting organic C and N abundance (e.g., N varying from 1.10% to 6.59%) and compositions (e.g., carboxylate functional group abundance varying from 13% to 21)^16^. After the resuspension of purified HAs in artificial groundwater amended with different radionuclides, partitioning coefficients (K_d_) of different radionuclides and their activity percentage were measured in the particulate (>0.45 µm), colloidal (3 kDa to 0.45 µm) and truly dissolved (<3 kDa) fractions of the HA-groundwater slurry. Size distribution of organic matter among these three fractions was also determined for the evaluation of their relationships with radionuclide partitioning parameters.

## Results

### Release of colloidal humic substances in HA-groundwater slurry

During the HA-groundwater resuspension experiment, certain amounts of organic macromolecules (i.e., colloidal organic matter, COM) were remobilized from the initial HAs and released into the groundwater. The results showed the proportion of released COM varied among different HAs (Table S2), ranging from 6% to 77% for organic carbon and from 0% to 74% for organic nitrogen. Additionally, the organic carbon and organic nitrogen of the initial HA had similar tendencies to be associated with the colloidal fraction, i.e., 29 ± 22% of organic carbon and 31 ± 23% of organic nitrogen were released from initial HAs into the colloidal fraction (ANOVA, *p* > 0.05, *n = 14*). In general, after a one-week resuspension experiment, considerable amounts of organic matter still remained in the particulate HAs phase under mildly acidic conditions (pH~5.5).

### Radionuclide distribution in size-fractionated SOM

**Figure 1:**
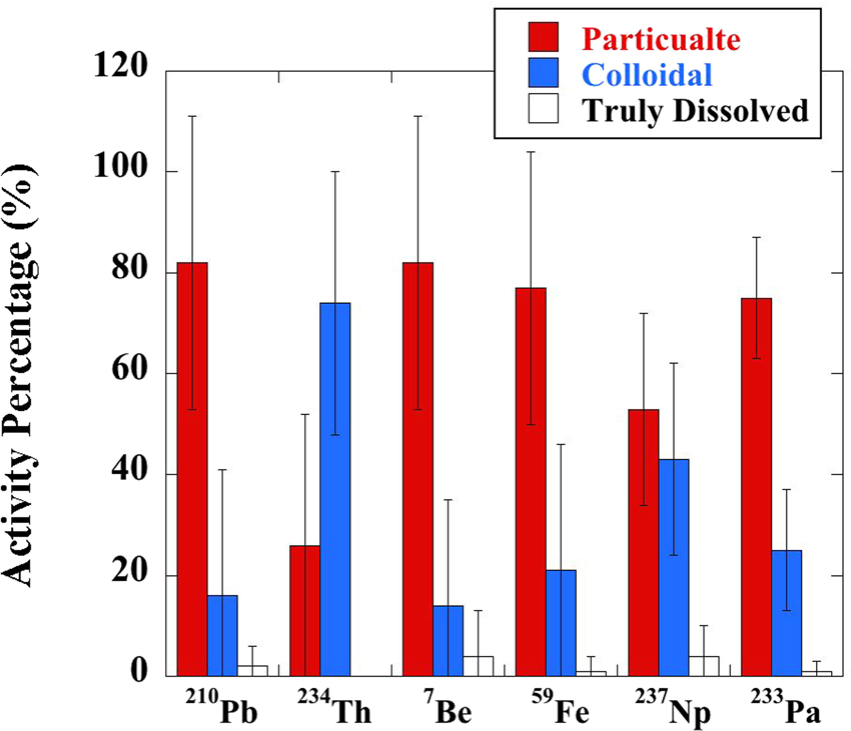
Averaged partitioning of six radionuclides among particulate (>0.45 µm), colloidal (3 kDa to 0.4 µm) and truly dissolved phases (<3 kDa) after the resuspension of HAs in groundwater.

Partitioning of different radionuclides among particulate (>0.45 µm), colloidal (3 kDa to 0.45 µm) and truly dissolved (<3 kDa) phases is shown in terms of activity percentage (Table 1) and plotted in terms of averaged values (Fig. 1). The activity percentage was calculated by the activity of radionuclide in each phase divided by the sum of radionuclide activity in all three fractions.

**Table 1:** Activity percentage of different radionuclides in particulate and colloidal phases after one-week HA-groundwater resuspension.

*Sample ID	Particulate activity percentage						Colloidal activity percentage					
	^210^Pb	^234^Th	^7^Be	^59^Fe	^237^Np	^233^Pa	^210^Pb	^234^Th	^7^Be	^59^Fe	^237^Np	^233^Pa
HA-1	91	32	89	84	51	78	8	67	10	16	48	22
HA-2	92	7	90	81	46	61	8	93	10	19	54	39
HA-3	99	26	99	97	72	85	1	74	1	3	27	15
HA-4	97	0	97	93	56	80	3	100	2	7	43	20
HA-5	90	38	89	83	54	75	10	62	11	17	45	25
HA-6	97	41	98	93	75	87	2	59	2	5	13	13
HA-7	91	17	88	74	50	76	9	83	12	26	50	24
HA-8	99	92	100	99	80	94	0	8	0	1	11	5
HA-9	98	46	100	98	72	78	2	54	0	2	23	16
HA-10	96	53	96	93	62	87	3	47	4	6	37	13
HA-11	15	0	13	16	18	61	74	99	69	77	78	39
HA-12	79	0	80	66	35	73	20	100	20	33	64	27
HA-13	9	0	10	12	14	51	79	100	59	79	65	49
HA-14	96	14	98	94	58	59	4	86	2	6	42	41
**AVG**	82	26	82	77	53	75	16	74	14	21	43	25
**STD**	29	26	29	27	19	12	25	26	21	25	19	12

*Detailed information for each HA sample ID can be found in Table S1.

“AVG” and “STD” denote the average values and standard deviation of all the HAs.

It is evident that different radionuclides showed distinct partitioning behavior among particulate, colloidal, and truly dissolved phases after HA-groundwater resuspension. Generally, ^210^Pb, ^7^Be, ^59^Fe and ^233^Pa were predominantly associated with the particulate HAs after one-week incubation, with an averaged activity percentage of 82 ± 29% for ^210^Pb, 82 ± 29% for ^7^Be, 77 ± 27% for ^59^Fe and 75 ± 12% for ^233^Pa (Fig. 1). In contrast, ^234^Th was primarily associated with the colloidal HAs (74 ± 26% on average), with a range from 47% in HA-10 to ~100% in some HA samples (e.g., HA-4). Unlike these radionuclides, ^237^Np activity was not concentrated within a given size fraction, instead it was evenly distributed between the particulate and colloidal phases, with an average for the 14 HA samples of 53 ± 19% in particles and 43 ± 19% in colloids (Fig. 1). Nevertheless, for all the six particle-reactive radionuclides, their activity concentrations in the truly-dissolved phase were consistently lower, <10% (Fig. 1).

The traditional partitioning coefficient (K_d_ value) was also used to describe the partitioning of different radionuclides between particulate (>0.45 µm) and dissolved phases (<0.45 µm) in natural waters^17–19^, which consider the colloidal fraction (3 kDa to 0.45 µm) as part of the liquid phase (the denominator of the K_d_ ratio; Equation 1). On the other hand, if the colloidal fraction is considered as part of the solid-phase fraction (the numerator of the K_d_ ratio; Equation 2), the colloidal partitioning coefficient (K_dc_) value can be calculated to used to describe ratio of radionuclide bound to the particulate and colloidal fractions^15^. Therefore, the K_d_ and K_dc_ values in the present study were calculated following the equations:

1 $${{\rm{K}}}_{{\rm{d}}}={{\rm{A}}}_{{\rm{p}}}\times {[({{\rm{A}}}_{{\rm{c}}}+{{\rm{A}}}_{{\rm{d}}})\times {{\rm{C}}}_{{\rm{HA}}}]}^{-1}$$

2 $${{\rm{K}}}_{{\rm{dc}}}=({{\rm{A}}}_{{\rm{p}}}+{{\rm{A}}}_{{\rm{c}}})\times {{(A}_{{\rm{d}}}\times {{\rm{C}}}_{{\rm{HA}}})}^{-1}$$

where A_p_ is the activity of radionuclide in the particulate phase (>0.45 µm), A_c_ and A_d_ represent the activity of radionuclide in the colloidal (3 kDa to 0.45 µm) and truly dissolved phases (<3 kDa), respectively, and C_HA_ is the concentration of added HAs (in kg/L). To ease presentation, K_d_ or K_dc_ values (in mL/g or L/kg) are reported in logarithmic form, logK_d_ and logK_dc_, respectively.

**Figure 2:**
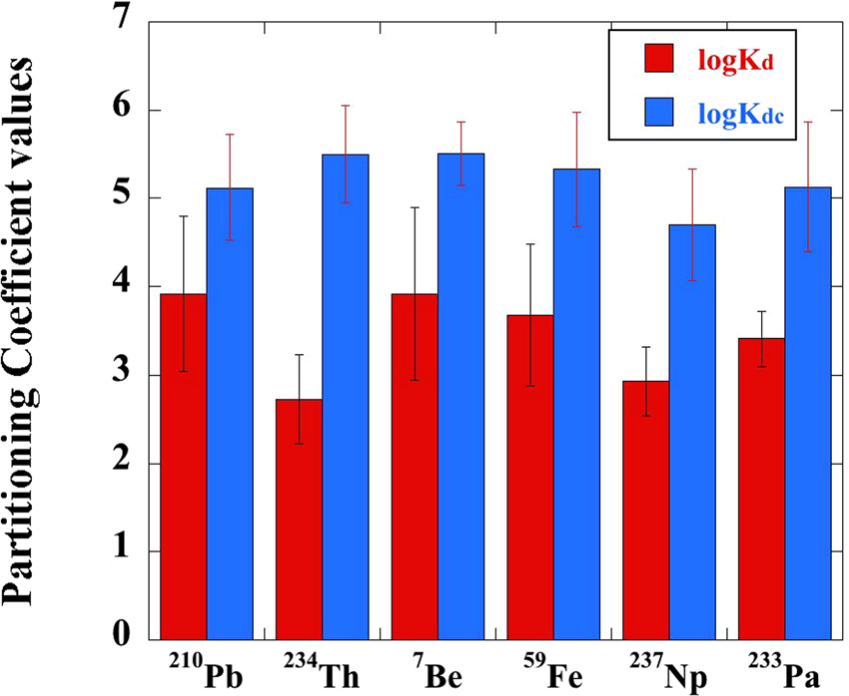
Averaged partitioning coefficient values of six radionuclides between particulate (>0.45 µm) and dissolved phases (<0.45 µm), logK_d_, and the averaged partitioning coefficient values of Pu between surface-bound fraction (>3 kDa) and truly dissolved phase (<3 kDa), logK_dc_, after mixing with HAs in artificial groundwater for one week.

For logK_dc_, in which the colloidal phase is considered as part of the solid-phase fraction (>3 kDa; Equation 2), they were generally twice greater than the logK_d_ values (>0.45 µm; Equation 1) (Table 2, Fig. 2). For example, ^7^Be had an averaged logK_d_ value of 3.92 ± 0.98, while its logK_dc_ reached values as high as 5.51 ± 0.26 (Table 2). Stated slightly differently and perhaps more poignantly, the K_dc_ value of ^7^Be is 39 times greater than the ^7^Be K_d_ value, demonstrating that how we assign the potentially mobile colloidal fraction in our conceptualization of radionuclide partitioning is very important. Additionally, most radionuclides, except ^234^Th, exhibited a wide range of both partitioning coefficient values. For example, they showed a logK_d_ range from 1.91 in HA-13 sample to 4.99 in the HA-3 sample for ^210^Pb and a logK_dc_ range from 3.76 in HA-13 sample to 5.72 in HA-4 sample. For ^234^Th, the logK_d_ values were consistently low compared to other radionuclides, due to the high activity in the colloidal HAs, with a relatively narrow range of values (2.10 in HA-2 sample to 3.99 in the HA-8 sample). Only four logK_dc_ values were obtained due to the fact that the activity in the truly dissolved phase (<3 kDa) was undetectable for most of HAs. Similar examples were also found for logK_dc_ values of ^7^Be and ^233^Pa.

**Table 2:** Partitioning coefficient values of different radionuclides on HAs.

Sample ID^2^	logK_d_ values^1^						logK_dc_ values^1^					
	^210^Pb	^234^Th	^7^Be	^59^Fe	^237^Np	^233^Pa	^210^Pb	^234^Th	^7^Be	^59^Fe	^237^Np	^233^Pa
HA-1	3.91	2.53	3.80	3.59	2.90	3.43	5.21	5.45	4.82	5.16	5.41	5.58
HA-2	3.94	2.10	3.83	3.52	2.81	3.10	5.52	—	—	5.91	5.07	5.51
HA-3	4.99	2.92	5.21	4.35	3.30	3.64	5.55	—	5.53	5.57	4.82	5.69
HA-4	4.36	—	4.47	4.00	2.99	3.51	5.72	—	5.82	5.71	4.99	—
HA-5	3.84	2.68	3.81	3.56	2.96	3.36	5.36	5.94	5.68	—	5.18	—
HA-6	4.43	2.72	4.57	4.04	3.37	3.72	4.99	—	—	4.78	3.76	—
HA-7	3.90	2.18	3.73	3.33	2.89	3.40	5.38	6.00	5.71	5.67	5.28	—
HA-8	4.98	3.99	5.23	5.03	3.48	4.11	5.10	—	—	5.63	3.88	4.54
HA-9	4.50	2.80	—	4.59	3.31	3.45	5.25	—	—	5.65	4.25	3.74
HA-10	4.29	2.95	4.24	4.05	3.11	3.70	5.24	—	—	5.55	4.83	—
HA-11	2.12	—	2.06	2.16	2.23	3.08	3.80	4.62	—	4.02	4.25	—
HA-12	3.47	—	3.48	3.18	2.62	3.33	5.02	—	—	5.93	5.50	5.69
HA-13	1.91	—	1.92	2.03	2.09	2.90	3.76	—	—	3.94	3.46	—
HA-14	4.25	2.47	4.59	4.05	3.02	3.08	5.81	—	—	5.80	5.10	—
AVG	3.92	2.73	3.92	3.68	2.93	3.41	5.12	5.50	5.51	5.33	4.70	5.13
STD	0.88	0.50	0.98	0.80	0.39	0.31	0.60	0.55	0.36	0.65	0.63	0.74

^1^See the definition in Equation 1 and 2.

“—” denotes data not available due to the undetectable activity in particulate (>0.45 µm) or truly dissolved phases (<3 kDa).

^2^Detailed information for each HA sample ID can be found in Table S1.

“AVG” and “STD” denote the average values and standard deviation of all the HAs.

## Discussion

With widely contrasting soil origins and different molecular and elemental compositions, it is evident that different HAs have a distinct influence on the partitioning of individual radionuclides among particulate, colloidal, and truly dissolved fractions, except for Th. In the HA-groundwater slurry, Th exhibited a relatively narrow range of partitioning coefficients, e.g., logK_d_ values, with many logK_dc_ values missing because of mostly undetected truly-dissolved Th concentrations. Furthermore, the characteristic feature of Th was that the vast majority of it was partitioned into the colloidal phase (74 ± 26%, Fig. 1), regardless of the soil type from which the HA was purified. Thus, such strong partitioning of Th with colloids can be one of the reasons why the Th isotopes were rarely applied as the tracers for soil erosion in natural environments even though it is a strongly particle-reactive metal, as significant redistribution of particulate Th isotopes into a mobile colloidal phase might occur and the initially Th-traced soil source would be lost during a strong rainfall or storm event. Under this condition, the determined mobility of soil particles using Th isotopes would be overestimated due to the more mobile colloidal Th. For example, this observation is consistent with the observation in a ^232^Th-contaminated wetland on the Savannah River Site in South Carolina in which ^232^Th migrated more than 100 times further than the expected migration distance due to the presence of a mobile organic-^232^Th fraction that accounted for 33% of the soil (not HA) ^232^Th_(aq)_^20^. Related to this, previous studies of Pu, which commonly exists in the +4 oxidation state like Th, indicated that Pu showed similar partitioning behavior to HA as Th (e.g., 76 ± 13% Pu existed in the colloidal phase^21^). This implies that strong natural or anthropogenic flooding events may also affect the validation of fallout ^239,240^Pu as a tracer of soil erosion in natural soil environments^22,23^ due to colloidal Pu remobilization from soil particles.

In contrast to Th, residual particulate HAs had a stronger affinity to most of the other radionuclides (i.e., Pb, Pa, Be and Fe) relative to the released colloidal HAs after one-week of HA-groundwater resuspension (Fig. 1). This partitioning behavior to HAs suggests that a majority of these radionuclides will remain fixed to soil particles even during strong flooding events. Minor repartitioning of these five radionuclides into the colloidal phase demonstrates that these radionuclides in natural soil environments have relatively higher stability in soil particles and less possibility of redistribution and exchangeability with surrounding waters or released COM from SOM during rainwater events and/or groundwater movement. Thus, the release of COM and strong natural or anthropogenic soil erosion events have minor impact on the validity of Pb, Pa, Be and Fe isotopes as the tracers of soil particles in natural environments. The widely used naturally occurring ^210^Pb and ^7^Be, as well as the artificial radionuclide, ^59^Fe, can serve as suitable tracers for assessing soil erosion, movement and accumulation in natural environments^4,6^, although Pa isotopes are rarely used as the tracers for soil erosion.

In contrast to the above particle-reactive radionuclides that can serve as tracers for soil erosion, Np in natural oxic environments mostly exists in the oxidation state of Np(V) (i.e., in the NpO_2_^+^ form) and exhibits a relatively high aqueous solubility (usually found as a low-molecular-weight species in the <3 kDa fraction) and thus would be expected to exhibit greater mobility in natural soil environments^24,25^. Therefore, Np radioisotopes are rarely used in the soil/particle-tracing research field. However, as a by-product of nuclear reactors and plutonium production, it is common to detect ^237^Np in radioactive waste in surrounding areas of nuclear processing facilities^26–28^. Thus, our results could provide new insights into the partitioning behavior of artificial ^237^Np during soil erosion in radionuclide-contaminated sites. Basically, our results indicate that over 90% of Np, on average, was fixed by either particulate or colloidal HAs after one-week in a HA-groundwater resuspension (Fig. 1 and Table 1). However, a certain portion of the added Np still remained in the truly dissolved phase, although still lower than 10% on average (Table 1). Such activity concentrations of Np in the truly dissolved phase would be inconsistent with a +4 cation, as evidenced by Th(IV) partitioning. While we did not measure the Np oxidation state of the truly dissolved fraction, it appears that this fraction remained in the +5, neptunyl form. Related, Kaplan *et al*.^29^ showed that soil bound Pu was almost exclusively +4, whereas the aqueous Pu was almost exclusively +5, (PuO_2_^+^) in a pH 5.5 soil-groundwater resuspension experiment. The partial partitioning of Np to the particulate or colloidal fractions is likely attributable to the HA substances capable of reducing the Np(V) to Np(IV) that has a relatively much stronger particle-reactivity^30,31^. Np(IV) has been shown to be complexed with organic matter, whereas Np(V) should have only a low tendency to partition to organic matter and would remain primarily in the truly dissolved phase^25^. Therefore, even though the artificial ^237^Np in radioactive waste may initially have strong mobility when it is released into the soil environments, humic acid substances that are ubiquitously present in natural soils would favor the fixation of ^237^Np by potentially immobile particles via reduction of readily-dissolved ^237^Np(V) to particle-reactive ^237^Np(IV). Although there still was considerable ^237^Np binding to potentially mobile colloidal and truly dissolved organic matter, humic acid substances could partially retard the movement of ^237^Np in polluted environments and reduce its movement and radiation risk from Np to the surrounding areas.

Similar to Np, particle-reactive Fe and Pa are also redox-sensitive elements in natural environments, existing as Fe(III)/Fe(II) and Pa(V)/Pa(IV), respectively, depending on redox conditions. Therefore, accompanying the reduction of Np(V) to Np(IV), the reduction of more particle-reactive Fe(III) to more readily-dissolved Fe(II) was possible during the HA-groundwater resuspension. However, the consistent predominance of particulate Fe as mentioned above (Fig. 1) may indicate only minor or no reduction of Fe(III) occurred during the incubation experiment. Such a difference between Fe and Np could be related to the fact that Fe(II) from HA reduction can be rapidly oxidized back to Fe(III) by O_2_ under oxic conditions (i.e., redox cycling), especially when the pH is higher than 5 ^32^, while this may not happen to Np. For Pa, since it primarily existed as Pa(V) in oxic conditions and Pa(IV) typically has stronger particle-reactivity than Pa(V), the high activity of Pa in the particulate phase (i.e., strong Pa fixation by potentially immobile particles) may indicate HA-derived reduction of Pa during one-week incubation. The partitioning behavior of Pa was very different from that of Np, even though both radionuclides are most stable in the natural environment in the +5 oxidation state. This difference was attributed to the fact that Np(V) has very weak particle-reactivity while Pa(V) still has considerable particle-reactivity even though it is weaker compared with Pa(IV)^33^. The Pa sorbed strongly to the HA with undetectable activity in the truly dissolved fraction (Fig. 1), consistent with a tetravalent cation, whereas the Np still had the minor fraction associated with the truly dissolved fraction (Fig. 1), consistent with the +5 oxidation state.

**Figure 3:**
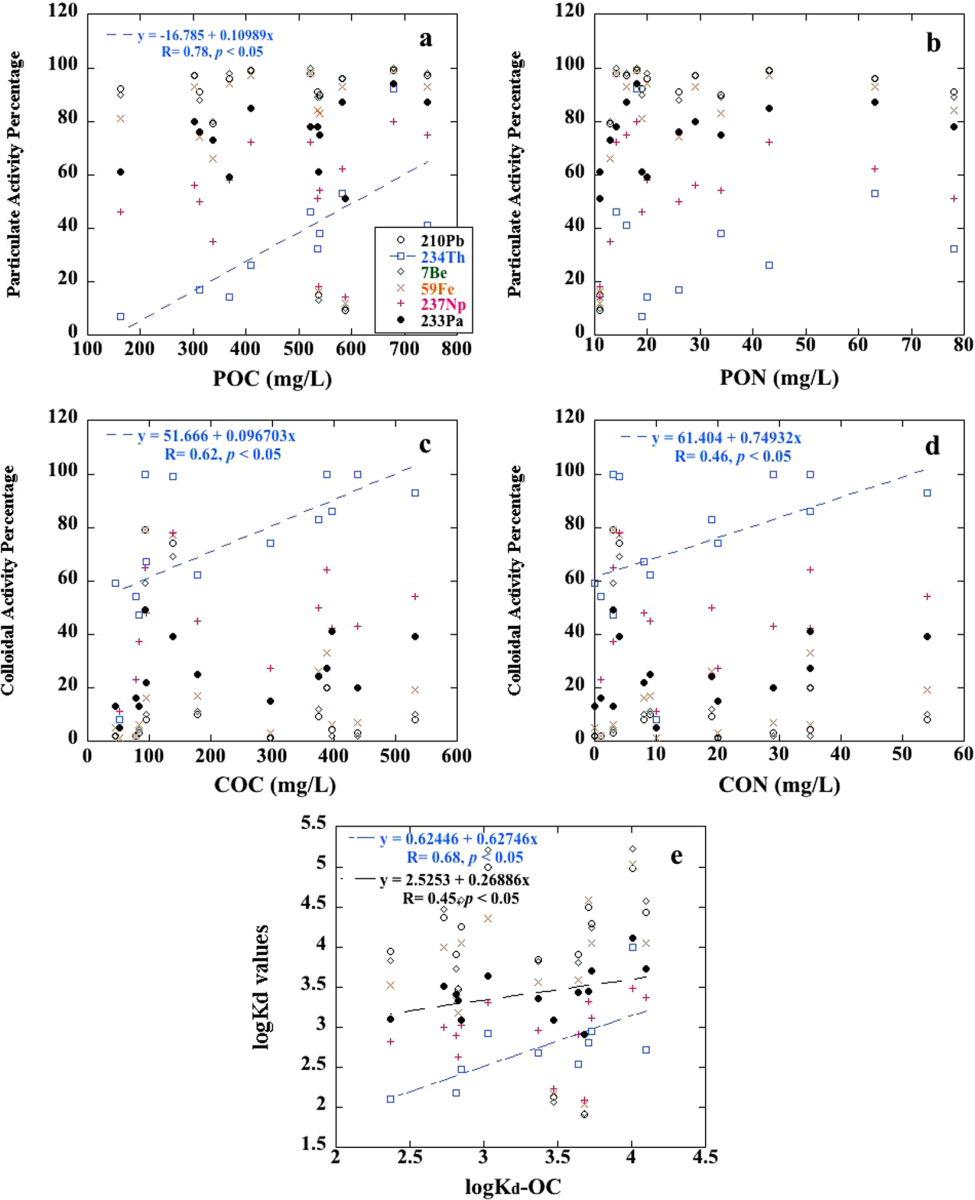
Correlation between the particulate activity percentage of radionuclides and the concentrations of (**a**) particulate organic carbon (POC) and (**b**) organic nitrogen (PON), between the colloidal activity percentage of radionuclides and the concentrations of (**c**) colloidal organic carbon (COC) and (**d**) organic nitrogen (CON), and (**e**) between the particulate-dissolved partitioning coefficient values (logK_d_) for different radionuclides and the particulate-dissolved partitioning coefficient values of organic carbon (logK_d_-OC) in the HA-groundwater slurry. One-tailed *p* values were presented for linear regression, and the concentrations on the x-axis were derived from the 14 HA samples described in Tables 1 and 2.

The differences noted in the partitioning behavior of the radionuclides to the particulate, colloidal, and truly dissolved fractions are attributed to differences in the inorganic chemical properties of the radionuclides and the differences in the organic compounds comprising the particulate and colloidal fractions. The 14 HA samples used in this study were analyzed for total carbon and total nitrogen content and for moiety composition by solid state ^13^C nuclear magnetic resonance (NMR) spectroscopy (details are presented in Table S1). As shown in Fig. 3c,d, a significant positive relationship is observed between the colloidal Th fraction and the concentrations of colloidal organic carbon and nitrogen (COC and CON in units of mg/L). Activity percentage of particulate Th was only positively correlated with the concentrations of particulate organic carbon (POC, Fig. 3a), not with particulate organic nitrogen (PON, Fig. 3b). In terms of partitioning coefficients, strong positive correlations between the Th K_d_ values and the organic carbon partitioning between particulate and dissolved phases (i.e., logK_d_-OC, whereby the POC concentration, divided by the total mass concentration of added HA, and the sum of COC and truly dissolved organic carbon concentrations, Fig. 3e) was also found. Together, these observations all consistently suggest a tight association of Th isotopes with many but specific organic compounds during its adsorption onto soil particles (i.e., particulate Th) and its exchange with surrounding rainwater or groundwater (i.e., colloidal Th), as also observed in aquatic environments^19,34,35^.

In comparison, the activity percentage of other studied radionuclides did not exhibit a strong relationship (Fig. 3), demonstrating that the partitioning behavior of these radionuclides (i.e., Pb, Be, Fe, Pa and Np) during the HA-groundwater resuspension may be influenced by other mechanisms or individual organic functionalities (see discussion below). However, among these five radionuclides, the adsorption behavior of Pa on particulate HAs (i.e., logK_d_) appeared to be relatively more affected by the partitioning between particulate and colloidal organic carbon, as suggested by the positive correlation between Pa logK_d_ and the partitioning coefficient values of organic carbon between particulate and dissolved phases (i.e., logK_d_-OC, Fig. 3e), similar to the observation for Th (Fig. 3e). Nevertheless, different from Th showing a synchronous influence from POM and COM on its partitioning behavior, the binding of Pa to organic matter was more prevalent in the partitioning of Pa onto particles during the HA-groundwater resuspension, and the organic binding of Pa may be either relatively weaker, or more varied, when compared to Th interactions with organic matter (r = 0.45 for Pa vs. r = 0.68 for Th, Fig. 3e).

Since the HAs samples were also characterized at the molecular-level through solid state ^13^C NMR spectroscopy measurements, this allows us to further explore potential correlations between the uptake of different radionuclides and specific organic functional moieties. However, no significant correlations were found between the surface-bound partitioning coefficients of radionuclides (i.e., logK_dc_ values) and the total abundance of all detected organic ligands (i.e., particulate plus colloidal fractions), including alkyl, O-alkyl, aryl, O-aryl, COO and carbonyl-C associated compounds (data not shown). Since our sample amount after separation was not enough for the accurate measurement of organic ligands in particulate and colloidal fractions, the assumption was made to indirectly obtain the concentrations of particulate plus colloidal organic ligands concentrations. This allowed to obtain a relationship between radionuclides and particulate or colloidal ligands. The assumption is that these organic compounds in the initial HA have similar releasing ability to the colloidal phase as the bulk organic carbon pool does.

**Figure 4:**
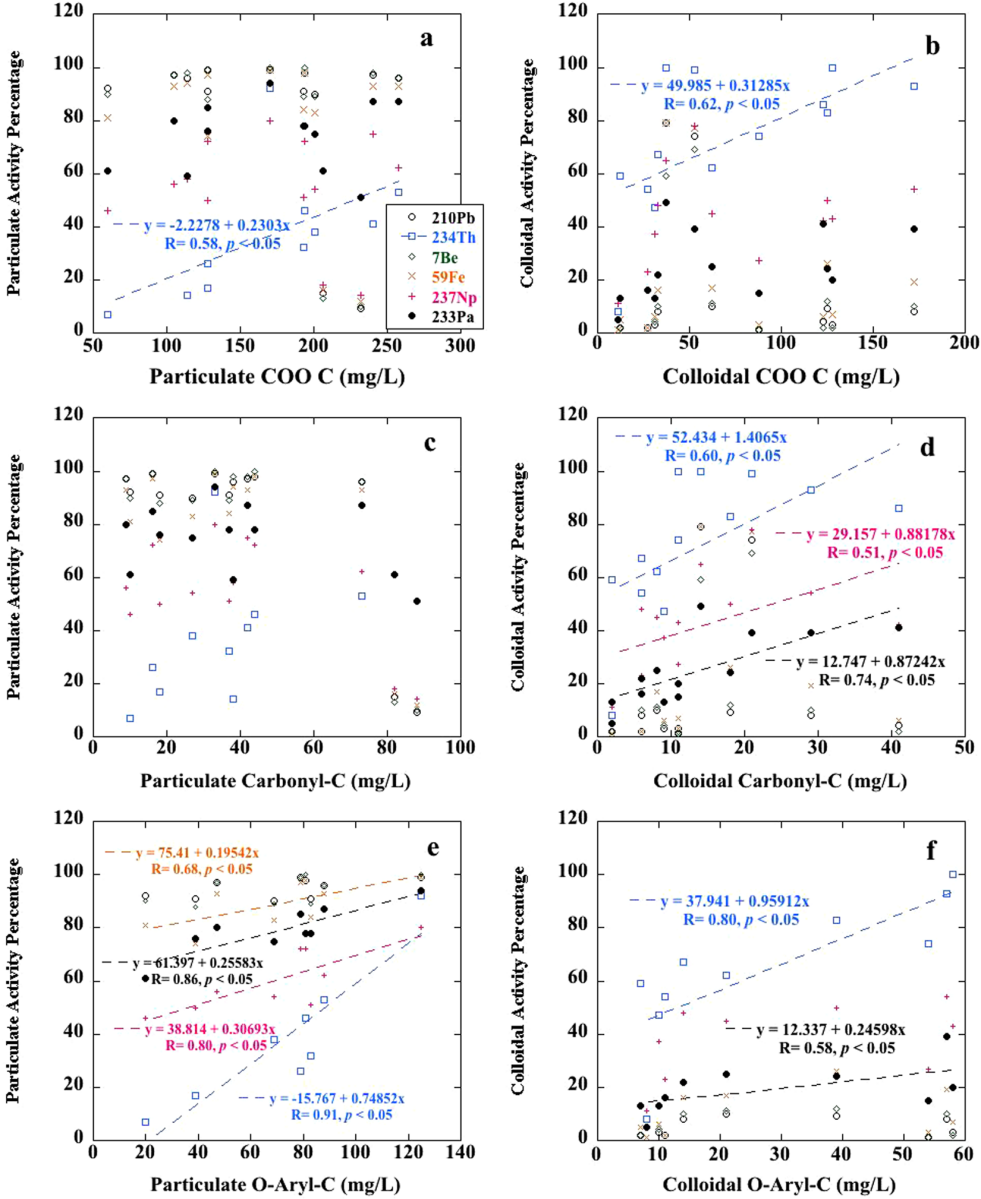
Correlation between the particulate/colloidal activity percentage of radionuclides and the concentrations of particulate/colloidal carboxylate-containing (COO C) organic compounds (**a** and **b**) and the concentrations of particulate/colloidal carbonyl-containing organic compounds (**c** and **d**), as well as the concentrations of particulate/colloidal O-aryl-containing organic compounds (**e** and **f**) in the HA-groundwater slurry. One-tailed *p* values were presented for linear regression, and the concentrations on the x-axis were derived from the 14 HA samples described in Tables 1 and 2.

The results showed different correlations between the particulate or colloidal radionuclide activity concentrations and the concentrations of particulate or colloidal carboxyl-, carbonyl- or O-aryl-containing organic moieties, respectively (Fig. 4). For example, a significant positive relationship was observed between Th and these three functional group-containing organic compounds, except the particulate carbonyl-containing moieties. In comparison, Pa did not show strong correlations with carboxylate compounds (Fig. 4a and b), but colloidal Pa had strong positive correlations with the abundance of carbonyl or O-aryl-containing compounds (Fig. 4d and f), in addition to the positive correlation with O-aryl functional groups in the particulate phase (Fig. 4e). In more details, the carbonyl and O-aryl groups can be taken as a proxy for the hydroquinone/quinone compounds^36,37^, a redox regulator commonly existing in humic substances^38^. Previous studies have provided some evidence^33,39^ for this, suggesting hydroquinone/quinones play an important role in the binding of Pa through redox coupled to chelation reactions. Therefore, it is also possible that the redox potential of particulate and/or colloidal hydroquinone/quinone (0.1 V to 0.3 V^40^) mediated the reduction of Pa(V) to Pa(IV), followed by binding of Pa(IV) to the O of the carbonyl or O-aryl functionality in the quinone compounds. Different from Pa, Th not only binds with hydroquinone/quinone compounds, but can also be associated with other carboxylate-containing moieties, such as hydroxamate, which are substituted amide siderophore compounds commonly present in the HAs and has been proposed to strong bind Pu, a Th-like actinide metal^41,42^.

Additionally, it was unexpected to find a significant positive relationship between the activity percentage of colloidal Np and the concentrations of colloidal carbonyl functionalities (Fig. 4d), as well as between Np and O-aryl-containing compounds in the particulate phase (Fig. 4e). To our knowledge, this is the first evidence that carbonyl-containing organic compounds may be responsible for Np complexation by colloidal organic matter. This complexation may occur through the reaction with colloidal hydroquinone/quinone compounds, similar to Pa. Hydroquinones have been documented to reduce Np to its tetravalent state (Np(IV))^38,43^. Similarly, a portion of Np(IV) was also directly bound to the O-aryl functionalities of particulate hydroquinones compounds after its reduction, leaving minor unreduced Np(V) in the truly dissolved phase (<3 kDa) as unbound ionic Np(V). Therefore, together with the partitioning of Np among particulate, colloidal and truly dissolved phases (Figs 1 and 2), it is hypothesized that during natural or anthropogenically induced soil erosion events (e.g., intense flooding and base injection remediation), the low-molecular-weight or ionic Np(V) in soil environments would be firstly reduced to particle-reactive Np(IV) through the reaction with the released colloidal hydroquinone or other organic compounds from HAs, followed by chelating with other colloidal functional groups making Np potentially mobile in the colloidal form. In contrast, binding with particulate hydroquinone compounds in residual soil particles would render Np immobile. When assessing potential radiation risks to the surrounding areas from artificial ^237^Np, its abundance in more mobile colloids would largely depend on how much carbonyl- or O-aryl-containing organic compounds (a proxy of the redox product from hydroquinone) would be released from the soils during soil erosion events.

For other non-actinide metal radioisotopes (i.e., Pb, Fe and Be) serving as the tracers of soil particle movement, only Fe was found to have significant a correlation with O-aryl functionalities in the particulate fraction (Fig. 4e). Together with the predominance of particulate Fe after HA-groundwater resuspension (Fig. 1), this suggested that most of Fe was directly bound to the O of particulate O-aryl-containing organic compounds, like the Np and Pa association with particulate hydroquinones, while the difference was that no reduction of Fe occured prior to its binding to particulate O-aryl-containing compounds. Although Fe has been documented to complex strongly with hydroxamate siderophores that typically contain carboxyl groups which are present in HAs, no correlation was found between Fe and carboxyl groups^33,39,44^ (or Pu, an actinide element similar in its ionic potential^41,42^ to that of Fe^3+^). The lack of relationships might have resulted from the fact that in mildly acidic conditions (pH = 5.5) Fe^3+^ may preferentially bind to O-aryl functionalities, rather than to carboxylate organic moieties. In comparison, no relationship to organic functionalities was found for both Pb and Be (Fig. 4), indicating that the partitioning behavior of these two particle-reactive radionuclides may be simply regulated by non-specific adsorption onto the particulate surfaces. Such uniform adsorption onto particles for Pb or Be, as well as their weak binding to colloidal organic compounds (Fig. 1) further confirmed the low possibility of Pb/Be repartitioning due to the loss of specific particulate components when they are applied as the tracers of soil erosion, accumulation and movement.

In summary, even though HAs were collected from widely contrasting soil types and origins, most of Pb, Be and Fe was taken up by particulate HAs, suggesting naturally occurring (i.e., ^210^Pb, ^7^Be) or artificial radioisotopes (e.g., ^59^Fe) can serve as suitable tracers for soil loss, erosion and accumulation in widespread natural soil environments. A similar case was found for Pa although its isotopes are rarely applied in the field of soil tracing research. Th had extremely high K_dc_ values (i.e., strong affinity to colloidal fractions) and the values were relatively similar for all 14 HA sources, explaining why the Th isotopes are rarely applied as the tracers for soil erosion in natural environments. Th uptake was likely related to its tight association with different organic moieties in both particulate and colloidal fractions (e.g., carboxylate, carbonyl and/or O-aryl moieties that ubiquitously exist in the HAs of natural soils). On the other hand, humic acid substances could partially retard the movement of readily-dissolved Np in polluted environments and reduce its movement and radiation risk to the surrounding areas. In the present study, our reported significant relationships between organic carbon/functional groups and the partitioning behavior of some radionuclides further demonstrates the essential role of natural soil organic matter in the uptake of radionuclides during their participation in geochemical processes in soil environments, such as Pa with carbonyl-containing compounds and Np with carbonyl moieties or hydroquinones/quinones. Importantly, this information was obtained from HAs with varying organic matter abundance and from widely contrasting soil origins. Nevertheless, more direct spectroscopic investigations of the molecular-level composition of HAs that are responsible for the binding and enrichment of radionuclides are still needed in future studies.

## Materials and Methods

### Purification of humic acid substances

Basically, the HAs from these soils were isolated and further purified according to an alkaline extraction method from the International Humic Substance Society (IHSS)^11,45^. Although one can always debate the best method of extracting representative SOM^46^, we followed a standard protocol recommended by IHSS^47^.

Briefly, the dried soil was pre-treated with a 1 M HCl solution to separate the supernatant (i.e., fulvic acid) from the soil, followed by the addition of 0.1 M KOH under N_2_ purging and 0.3 M K^+^, as KCl. Then, the supernatant was acidified to pH of 1.0 using 6 M HCl to precipitate HAs, which were pelleted by centrifugation and then suspended in a 0.1 M HCl/0.3 M HF solution overnight for five times to minimize ash content. After HF digestion, Milli-Q water was used to wash HA with the purpose of minimizing ions. The abundance of organic C and N in HAs was determined using an elemental analyzer, and their molecular composition was chemically characterized by solid state ^13^C nuclear magnetic resonance (NMR) spectroscopy. All HA samples for the HA-groundwater resuspension experiments described below were stored frozen at −5 °C. HA characterization data were previously reported elsewhere^16^ and on IHSS website (http://humic-substances.org/13c-nmr-estimates-of-carbon-distribution-in-ihss-samples/).

### HA-groundwater resuspension experiment

To mimic the mildly acidic pH of 5.5, the average pH of natural rainwater and also the background groundwater pH at the Savannah River Site (SRS), USA^48,49^, the artificial groundwater, with low salt concentrations (<10^−2^ M) and minor abundance of carbonate and organic matter, was prepared as the medium for the HA resuspension experiments. The HA-groundwater suspension batch experiments were conducted basically similar to a previously reported procedure^15,21^. In brief, 5–6 mg of the purified HAs were pre-equilibrated in artificial groundwater in the centrifuge tubes for 48 h at room temperature (20 °C) to reach the dissolution equilibrium, since the HAs were extraceted under pH < 1 but the pH of artificial groundwater is 5.5. Then, ~50 Bq of each gamma emitting radionuclide, including ^234^Th, ^237^Np-^233^Pa, ^210^Pb, ^7^Be and ^59^Fe was added to the HA-groundwater slurry to a final volume of 4 mL. A relatively high HA concentration in the soil-water system was used in the laboratory experiment to mimic the soil erosion in natural soil environments. During the soil erosion and runoff events, the pond discharge or rainwater passes through surface soil environments, where organic-rich soils predominate and the HA concentration is high in the slurry system. The ^234^Th tracer was milked and purified from a ^238^U solution, while a ^237^Np-^233^Pa equilibrium solution (Pacific Northwest National Laboratory) was used directly as the tracer. ^210^Pb and ^210^Po were both purchased from Eckert & Ziegler Isotope Products, and the ^7^Be solution was obtained from the Paul Scherrer Institute, Switzerland. It should be noted that the tracer cocktail was amended with non-complexing PIPBS (piperazine-N,N”-bis(4-butane sulfonic acid); GFS chemicals, Cat 2360) buffer (pH of 5.5) in order to neutralize the acidic tracer and maintain this pH during the incubation period.

The radiolabeled HA-groundwater slurry was then mixed continuously for 7 days in the dark with an end-over-end mixer to ensure that quasi-equilibrium was attained^15^. In addition to this duplicate radiolabeled samples, two control samples were also carried out simultaneously, including 1) a radiotracer control without the HAs to monitor the radionuclide loss due to adsorption to labware including centrifuge tubes and filters, and 2) a HA control without the addition of radiotracers to monitor organic matter released from the HAs over the course of the experiment, which is for the measurement of size distribution of organic matter after the resuspension. After one-week period, particulate (>0.45 µm), colloidal (3 kDa to 0.45 µm), and truly dissolved (<3 kDa) phases were size fractionationated by using 0.45 µm centrifugal filter tubes followed by ultrafiltration with 3 kDa Microsep centrifugal filter tubes (Millipore). Each fraction was collected for the measurement of radionuclide activity and organic matter concentrations.

### Determination of radionuclides activity

To avoid geometry corrections, all the size fractions, including the particulate, colloidal and truly dissolved phases were corrected to the same volume and geometry for the counting of ^234^Th, ^233^Pa, ^237^Np, ^210^Pb, ^7^Be and ^59^Fe activity concentrations by a Canberra ultrahigh purity germanium well gamma detector at the decay energies of 63.5 keV, 312 keV, 86.5 keV, 46.5 keV, 477.6 keV and 1069 keV, respectively. Additionally, it should be noted that all the data were decay corrected to the same date, and that the ingrowth of ^233^Pa from ^237^Np decay was also corrected.

Mass balance of different radionuclides during the overall procedure was also monitored, basically showing that over 85% of all the radionuclides to be recovered although this varied for different radionuclides. To normalize and minimize the influence of different recoveries on the partitioning data of various radionuclides, the radionuclide which was lost onto the labware in the present work was considered to not participate in the experiment and was excluded from the calculation of activity percentage in various fractions and the calculation of partitioning coefficient values. This approach had been previously verified^50^.

### Measurement of organic carbon and nitrogen

Concentrations of organic carbon and nitrogen in the colloidal and truly dissolved fractions were determined with using a high temperature combustion method^51^ using a Shimadzu TOC-L analyzer. The organic carbon and nitrogen concentrations in the particulate phase of the HA-groundwater suspension were calculated as the difference between the total carbon/nitrogen contents of the added HAs and the sum of colloidal and truly dissolved phases.

## Electronic supplementary material

Supplementary Information
