# Supplementary material for: Radionuclide uptake by colloidal and particulate humic acids obtained from 14 soils collected worldwide
Source: Sci Rep. 2018 Mar 19;8:4795. doi: 10.1038/s41598-018-23270-0 (PMC5859050; doi:10.1038/s41598-018-23270-0)
Supplement: Supplementary file 1 — Supplementary Information [file 41598_2018_23270_MOESM1_ESM.pdf]

Supplementary Materials

Radionuclide uptake by colloidal and particulate humic acids obtained from 14 soils collected worldwide

Peng Lin[1]*, Chen Xu[1], Wei Xing[1], Luni Sun[1], Daniel I. Kaplan[2], Nobuhide Fujitake[3], Chris M. Yeager[4], Kathleen A. Schwehr[1], Peter H. Santschi[1]

[1] Department of Marine Science, Texas A & M University at Galveston, Galveston, Texas 77553, United States

[2] Savannah River National Laboratory, Aiken, South Carolina 29808, United States

[3] Division of Agroenvironmental Biology, Graduate School of Agriculture Science, Kobe University, Kyoto, 606-8501, Japan

[4] Los Alamos National Laboratory, Los Alamos, New Mexico 87545, United States

*Corresponding Author: Peng Lin (pengl1104@tamug.edu)

For submission to *Scientific Reports*

Supporting Information Contents: 3 pages. 2 tables

Table S1 Characterization of humic acid substances (HAs) derived from different soils in the present study. Data of HA1 to HA10 are published elsewhere[16], and HA11 to HA14 samples are from International Humic Substance Society (IHSS) (http://humic-substances.org/13c-nmr-estimates-of-carbon-distribution-in-ihss-samples/).

	Basic Information				Elemental Analysis (%)		Carbon-13 NMR (%)[1]				
**No.**	**HA ID**	**Site**	**Soil Order**	**Land use**	**C**	**N**	**Alkyl C**	**O-Alkyl C**	**Aryl C**	**O-Aryl C**	**COO C**
1	Palana	Brazil	Luvisols	Forest	50.40	6.59	20.4	31.9	19.5	7.5	17.4
2	Valence Lake	Hungary	Chernozems	Arable land	55.58	5.69	21.2	21.2	30.7	5.9	18
3	Galijembe	Tanzania	Luvisols	Arable land	56.57	4.83	11.3	22.8	36.7	10.3	16.8
4	Karcag	Hungary	Chernozems	Arable land	59.25	4.50	9.7	12	51.4	8.1	17.3
5	McCauly	Scotland	Podozol	Arable land	57.40	3.32	23.8	17.3	29.1	7	20
6	Oginosen	Japan	Andisols	Forest	63.00	1.20	5.5	6	56.8	10.9	17.5
7	Gyosei	Japan	Andisols	Glassland	55.00	3.46	7.2	19.4	46.3	6	18.4
8	Ichijima	Japan	Histosols	Glassland	58.40	2.15	16.9	21.5	35.9	10.3	13
9	Keiro-zan	Japan	Cambisols	Forest	48.10	1.18	22.4	26.1	23.8	7.1	16.6
10	Yamashiro	Japan	Cambisols	Forest	53.40	5.04	22.4	23.3	21	7.6	19.4
11	Suwannee River	U.S.A.	-	-	52.47	1.10	-	-	-	-	20
12	Pahokee Peat	U.S.A.	-	-	56.84	3.74	-	-	-	-	-
13	Nordic Lake	U.S.A.	-	-	53.17	1.10	-	-	-	-	21
14	Elliott Soil	U.S.A.	-	-	58.13	4.14	-	-	-	-	18

"-" denotes data not available.

Table S2 Concentrations and percentage of organic carbon and nitrogen in the particulate and colloidal fractions after one-week HAs-groundwater resuspension.

Sample ID	POC (mg/L)	PN (mg/L)	COC (mg/L)	CON (mg/L)	%POC	%PN	%COC	%CON
HA-1	535	78	95	8	85	91	15	9
HA-2	163	19	532	54	23	26	77	74
HA-3	410	43	297	20	58	69	42	31
HA-4	303	29	438	29	41	51	59	49
HA-5	540	34	178	9	75	80	25	20
HA-6	743	16	45	0	94	100	6	0
HA-7	313	26	375	19	45	58	55	42
HA-8	679	18	51	10	93	64	7	36
HA-9	523	14	79	1	87	90	13	10
HA-10	583	63	84	3	87	96	13	4
HA-11	537	11	139	4	79	74	21	26
HA-12	338	13	388	35	47	28	53	72
HA-13	588	11	94	3	86	78	14	22
HA-14	369	20	397	35	48	36	52	64
